# A Case of Intestinal Obstruction with Unusual Features

**Published:** 1909-08-07

**Authors:** 


### SURGERY.

### A CASE OF INTESTINAL OBSTRUCTION WITH UNUSUAL FEATURES.

The treatment of appendicitis has been discussed so thoroughly during recent years that the subject has become a hackneyed one: and the majority of surgeons are now in agreement that an operation is necessary as soon as the symptoms of an acute attack have declared themselves. In saying this the writer refers to those cases in which the appendix is obviously acutely inflamed, and peritonitis or abscess formation has supervened. He is not concerned with the debated question as to whether a catarrhal attack should be allowed to subside and the appendix removed subsequently in the quiescent stage or an immediate operation should be performed.

Nevertheless one occasionally comes across cases in which the patient has recovered from a definitely acute attack of appendicitis with abscess, in which no operation has been performed, either deliberately or because the circumstances did not permit of a laparotomy being done, *e.g.* the sudden onset of an attack of acute appendicitis in a remote part of the country where there are no facilities for abdominal surgery.

The result is usually fatal, but the patients sometimes recover spontaneously; and such cases might be used as an argument in favour of expectant treatment, if one did not have the opportunity from time to time of following the after history of these patients. The case about to be described is an illustration of this. The patient, a stonemason, aged 54, presented himself with symptoms of intestinal obstruction.

The history he gave was as follows: In January of the present year he was taken ill with sudden acute abdominal pain, referred at first to the umbilicus, and after a few days to the right iliac fossa. He was put to bed and treated by a doctor, who told him he was suffering from appendicitis. He was treated with purges and the application of turpentine stupes to the abdomen. He was also given subcutaneous injections of morphia to diminish his pain. He was exceedingly ill, and for some days his life was despaired of, but eventually he began to recover, and in a fortnight he was considered to be out of danger. His doctor was not communicated with, and no information could be obtained as to his exact state at this time, *i.e.* what was his condition on abdominal examination. His convalescence was long and tedious, and he was kept in bed until the end of April. During this time grat difficulty was experienced in getting his bowels to act, and an evacuation was only obtained in response to calomel or castor oil.

He gradually recovered strength after getting up, but the difficulty with the bowels persisted, and he had occasional attacks of vomiting accompanied by abdominal distension. He exercised great care in the choice of his diet, but in spite of this he became suddenly worse on July 16, and from this date his constipation became absolute. The vomiting, which had been up till now occasional, became constant, and was made worse by any attempt to take food, which caused a sensation of "being blown up inside." The last material vomited before he was seen "smelt like a motion."

On examination he was an emaciated but not cachectic looking man, in obvious distress. His temperature was 96° F., and his pulse 120. He complained of great pain just below the umbilicus. The abdomen was distended, but not excessively so. The movements of the abdominal wall on respiration were limited: but this appeared to be rather a mechanical result of the distension than due to inflammation of the parietal peritoneum. There was no tenderness on palpation, but a general sense of resistance on the right iliac fossa. No mass was felt. The distension was of the small intestine pattern, *i.e.* the centre of the abdomen was prominent and resonant on percussion, but the flanks were not distended. Nothing abnormal was felt on digital examination of the rectum. Apart from the attack of appendicitis he had had no other abdominal illness, and there was no family history of carcinoma.

Immediate operation was decided upon. The patient was anæsthetised. He was first examined with the sigmoidoscope to exclude disease of the sigmoid. None was found. The abdomen was then opened by a vertical incision through the right rectus muscle, the centre of the incision being one inch below the umbilicus. Distended small intestine at once presented. It was seen to lead down to the right iliac fossa, where a portion of collapsed small gut was seen. A puncture was made in the distended small gut and the intestinal contents allowed to escape. The puncture was sewn up with Lembert sutures. In the neighbourhood of the cæcum the intestines were matted together in a dense mass of adhesions, some of which were as large as a man's thumb. These were ligatured and divided one by one and the small intestine gradually straightened out. No point of acute strangulation was found. The cæcum itself was firmly bound down to the posterior abdominal wall. The appendix was felt in the adhesions but could not be freed. The wound was sewn up, but the patient never rallied, and died six hours after the operation.

An autopsy was performed, and revealed further extensive adhesions in the pelvis, which were binding the sigmoid and rectum to the bladder. No evidence of carcinoma was found. It appeared that the patient must have had an enormous appendix abscess which had been entirely absorbed spontaneously.

The case was of more than usual interest in showing how large an abscess can be absorbed by natural processes, if the resistance of the peritoneum to infection by the bacillus coli is not impaired. At the same time it must be supposed that the appendix abscess was directly responsible for the patient's death. It is also presumable that if a free exit had originally been given to the pus by operation, the dense adhesions, which subsequently caused the intestinal obstruction, would not have formed, and so the patient's life might have been saved.

